# Pioneering fully robotic donor hepatectomy and robotic recipient liver graft implantation – a new horizon in liver transplantation

**DOI:** 10.1097/JS9.0000000000001031

**Published:** 2024-01-04

**Authors:** Dieter C. Broering, Dimitri A. Raptis, Yasser Elsheikh

**Affiliations:** Organ Transplant Center of Excellence, King Faisal Specialist Hospital and Research Centre, Riyadh, Saudi Arabia

We would like to report a landmark achievement in the field of liver transplantation from the King Faisal Specialist Hospital and Research Center in Riyadh, Saudi Arabia. Our team performed in August 2023 the world’s first successful fully robotic recipient hepatectomy and liver graft implantation derived from a fully robotic living donor hepatectomy. This is a novel approach employing state-of-the-art technology to further advance living donor liver transplantation.

In recent years, while the integration of minimally invasive surgical techniques has marked a significant advancement in the field of living donor hepatectomy, the majority of such procedures are still performed through the traditional open surgical approach^1^. At our center, we have performed over 1000 minimally invasive living donor hepatectomies and currently offering the robotic approach to all donors. Having mastered the technique and observed favorable results (data not shown) in the donor, the obvious next step was to offer this technology to the liver recipients, ensuring a higher precision and potentially minimizing the risk of complications.

In April 2022, a case report was published describing the first laparoscopic donor and recipient hepatectomy followed by robot-assisted liver graft implantation performed at the Seoul National University Hospital in South Korea^2^. Such procedures are named ‘hybrid’ when combining two or more different approaches within the same operation. Later, another news report was released in August 2023, where the team at Washington University’s School of Medicine in the United States of America successfully performed the first robot-assisted whole-liver transplant derived from a deceased donor. However, our reported technique unifies both the donor hepatectomy as well as the recipient hepatectomy and implantation processes under a single robotic approach, thereby moving beyond assisted or hybrid techniques to offer a complete robotic solution.

Following six fully robotic donor and *hybrid robotic* recipient liver graft implantations, we have now performed a total of three successful *fully robotic* recipient procedures without any donor or major recipient morbidity (Table 1). The crucial steps involve the utilization of robotic arms to facilitate meticulous dissection, enhancing safety and precision during the procedure. Furthermore, the robotic system provides a three-dimensional view of the operative field, offering a superior visualization and facilitating a finer manipulation of tissues compared to conventional techniques.

**Table 1:** Donor and recipient characteristics and outcomes of full robotic donor hepatectomy within this report

Parameters	Donor A	Donor B	Donor C
Donor characteristics			
Age, years	29	32	35
Sex	Male	Female	Male
BMI, kg/m^2^	20.4	27.6	26.8
Degree of relatedness	Third	Second	Second
Relation to recipient	Cousin	Daughter	Son
Donor operation characteristics			
Donor hepatectomy	Right lobe	Right lobe	Right lobe
Graft weight, g	680	590	665
Estimated liver remnant, %	32	42	38
Number of bile ducts	1	1	1
Number of hepatic arteries	1	1	1
Number of portal veins	2	1	1
Number of hepatic veins	1	1	2
Operation duration, min	460	355	431
Estimated blood loss, min	70	60	80
Donor postoperative characteristics			
INR day 1 postoperatively, ratio	1.9	1.3	2.0
INR day 3 postoperatively, ratio	1.7	1.4	1.7
Bilirubin day 1 postoperatively, μmol/l	63	19	34
Bilirubin day 3 postoperatively, μmol/l	59	18	51
AST day 1 postoperatively, μmol/l	170	146	196
AST day 3 postoperatively, μmol/l	103	74	106
ALT day 1 postoperatively, μmol/l	212	156	284
ALT day 3 postoperatively, μmol/l	155	111	190
Donor postoperative complications			
Complications of any severity	0	0	0
Major complication (Grade >2)	0	0	0
Hospital stay, days	4	4	4s
Follow-up, days	23	17	16
Parameters	Recipient A	Recipient B	Recipient C
Recipient characteristics			
Age, years	67	66	57
Sex	Male	Female	Female
BMI, kg/m^2^	27.3	25.5	27.0
Diabetes	Yes	Yes	No
Hypertension	Yes	Yes	No
Ascites	No	No	Yes
Jaundice	No	No	Yes
Splenomegaly	Yes	No	Yes
GRWR, %	0.81	0.98	1.10
Indication for transplantation	NASH	HCV cirrhosis	NASH
Hepatocellular carcinoma (HCC)	Yes	Yes	No
MELD-Na	10	8	14
HCC-MELD-Na	22	22	–
Recipient operation characteristics			
Operation duration, min	756	526	572
Estimated blood loss, ml	1000	700	1000
Blood units transfused, number	3	0	2
Recipient postoperative characteristics			
INR day 1 postoperatively, ratio	2.5	1.9	3.4
INR day 7 postoperatively, ratio	1.2	1.2	1.3
Bilirubin day 1 postoperatively, μmol/l	61	36	60
Bilirubin day 7 postoperatively, μmol/l	17	24	63
AST day 1 postoperatively, μmol/l	282	358	1080
AST day 7 postoperatively, μmol/l	48	106	66
ALT day 1 postoperatively, μmol/l	461	373	1457
ALT day 7 postoperatively, μmol/l	161	213	352
Recipient postoperative complications			
Acute cellular rejection	No	Yes	No
Complications of any severity	0	1	0
Major complication (Grade >2)	0	0	0
Comprehensive Complication Index	0.0	8.7	0.0
Intensive care unit (ICU) stay, days	4	3	4
Hospital stay, days	13	13	13
In-hospital mortality	No	No	No
Follow-up, days	23	17	16

ALT, alanine aminotransferase; AST, aspartate aminotransferase; GRWR, Graft-to-Recipient Weight Ratio; HCV, hepatitis C virus; INR, international normalized ratio; MELD-Na (Model for End-Stage Liver Disease – Sodium); NASH, nonalcoholic steatohepatitis.

Briefly, the robotic recipient surgical technique involves using five ports for the total hepatectomy phase. Typical Da Vinci Xi instruments^3^ used include the Fenestrated Bipolar, Vessel Sealer Extend, ProGrasp, and the Maryland Bipolar bipolar forceps, while Hem-o-lok^4^ and VesselSealer used for control of vessels. After complete mobilization of the liver and dissection of the hilum, a 12 cm Pfannenstiel incision is performed and a GelPort access system^5^ is applied. The recipient hepatic artery, portal vein, and bile duct are clamped using the Scanlan Reliance Bulldog Clamps^6^ and divided. The right, as well as the left/middle hepatic veins, are then divided with GIA (gastrointestinal anastomosis) vascular staples (Supplementary Figs 1–14, Supplemental Digital Content 1, http://links.lww.com/JS9/B644, Supplemental Digital Content 2, http://links.lww.com/JS9/B645). The liver is removed hand-assisted, and the right graft is inserted through the GelPort. The implantation phase starts with partial clamping of the inferior vena cava (IVC) just beneath the right hepatic vein staple line with a manually introduced, total intracorporeal Glover Clamp^7^ via the GelPort and the anastomosis is performed with the graft right hepatic vein in a continuous fashion using a Gortex 6/0 suture^8^. Finally, the recipient and donor right portal vein anastomosis is performed using a continuous Gortex 6/0 while the hepatic artery anastomosis with interrupted 7/0 or 8/0 Prolene sutures. The duct-to-duct anastomosis is performed with interrupted 7/0 PDS (polydioxanone suture) (Supplementary Figs 15–26, Supplemental Digital Content 2, http://links.lww.com/JS9/B645, Supplemental Digital Content 3, http://links.lww.com/JS9/B646, Supplemental Digital Content 4, http://links.lww.com/JS9/B647).

The robotic recipient total hepatectomy and right graft implantation currently do not come without potential challenges and limitations. Firstly, there are limitations regarding patient and graft selection. Currently, ideal candidates for robotic liver transplantation at our institution include recipients with a relatively low Model for End-Stage Liver Disease (MELD) score. The procedure is offered for right-only grafts due to the associated improved exposure they offer. Moreover, liver grafts less than 900 g as well as an anticipated Graft-to-Recipient Weight Ratio (GRWR) of more than 0.80%, are selected. From an anatomical perspective, the technique is currently applied when only single arterial and bile duct anastomoses are anticipated based on imaging, along with a single medial sector venous drainage, thus excluding any additional segment 5 or segment 8 hepatic venous anastomoses.

Technically, cirrhotic livers are stiff and more difficult to retract or mobilize. Sponges are used when retracting such livers to avoid injury and bleeding caused by the instruments. With regard to IVC clamping, the ergonomics of the Glover Clamp intended for open surgery is not ideal and we are currently working with the industry to develop strong and reliable fully robotic cava clamps. Another challenge is that the donor's right portal vein is relatively short, and to avoid tension to the anastomosis, sponges are placed behind the right graft to overcome this situation. Finally, an overall challenge with the robotic recipient approach is the limited exposure when compared to open surgery.

In addition to the highly specialized robotic donor hepatectomy, the requirement for the surgeon to perform both the donor hepatectomy and the recipient’s graft implantation robotically using the Da Vinci system^9^ is associated with prolonged operation durations compared to traditional open recipient transplantations. This, especially when performing two donor hepatectomies four times weekly at our institution, can contribute to significant fatigue for both the surgeon and the entire team, further exacerbating logistical challenges and imposing constraints on the collective operational capacity. To mitigate this, we recommend well-structured operation scheduling for the team.

As for the sustainability and reproducibility of our program, we believe it can be replicated in centers with similar expertise and resources. The key to sustainability lies in ongoing training, continuous refinement of techniques, and collaborative learning with other centers. Regular monitoring and adaptation of protocols based on feedback and outcomes are crucial.

We anticipate that this work will lead to advancements in the field of liver transplantation, marking a new era of enhanced safety and improved outcomes. We recommend reporting all unselected, consecutive donor as well as recipient cases in international living donor liver transplant registries, such as LDLTregistry.org^10^ following the IDEAL recommendations for surgical innovations^11^.

## Ethics approval

Ethics approval was obtained from the Institution Clinical Research Ethics Committee ID: RAC 2121012.

## Consent

Consent was obtained.

## Sources of funding

None.

## Author contribution

D.C.B., D.A.R., and Y.E.: report conception and study design, data acquisition, data analysis and interpretation, article drafting and approval.

## Conflicts of interest disclosure

The authors have no conflicts of interest.

## Research registration unique identifying number (UIN)

The data are derived from our institutional registry. This is a report. Does not require ClinicalTrials.gov or similar registration.

## Guarantor

Dimitri Raptis and Dieter C. Broering.

## Data availability statement

Majority of the data related to the reported cases are included in the manuscript table. However, we will share additional available data and results upon request from the corresponding author of the manuscript.

## Provenance and peer review

Not commissioned, externally peer-reviewed.

## Supplementary Material

SUPPLEMENTARY MATERIAL
