# Supplementary material for: Pioneering fully robotic donor hepatectomy and robotic recipient liver graft implantation – a new horizon in liver transplantation
Source: Int J Surg. 2024 Jan 4;110(3):1333–6. doi: 10.1097/JS9.0000000000001031 (PMC10942232; doi:10.1097/JS9.0000000000001031)
Supplement: SUPPLEMENTARY MATERIAL [file js9-110-1333-s001.pdf]

Pioneering Fully Robotic Donor Hepatectomy and Robotic Recipient Liver Graft Implantation – A New Horizon in Liver Transplantation

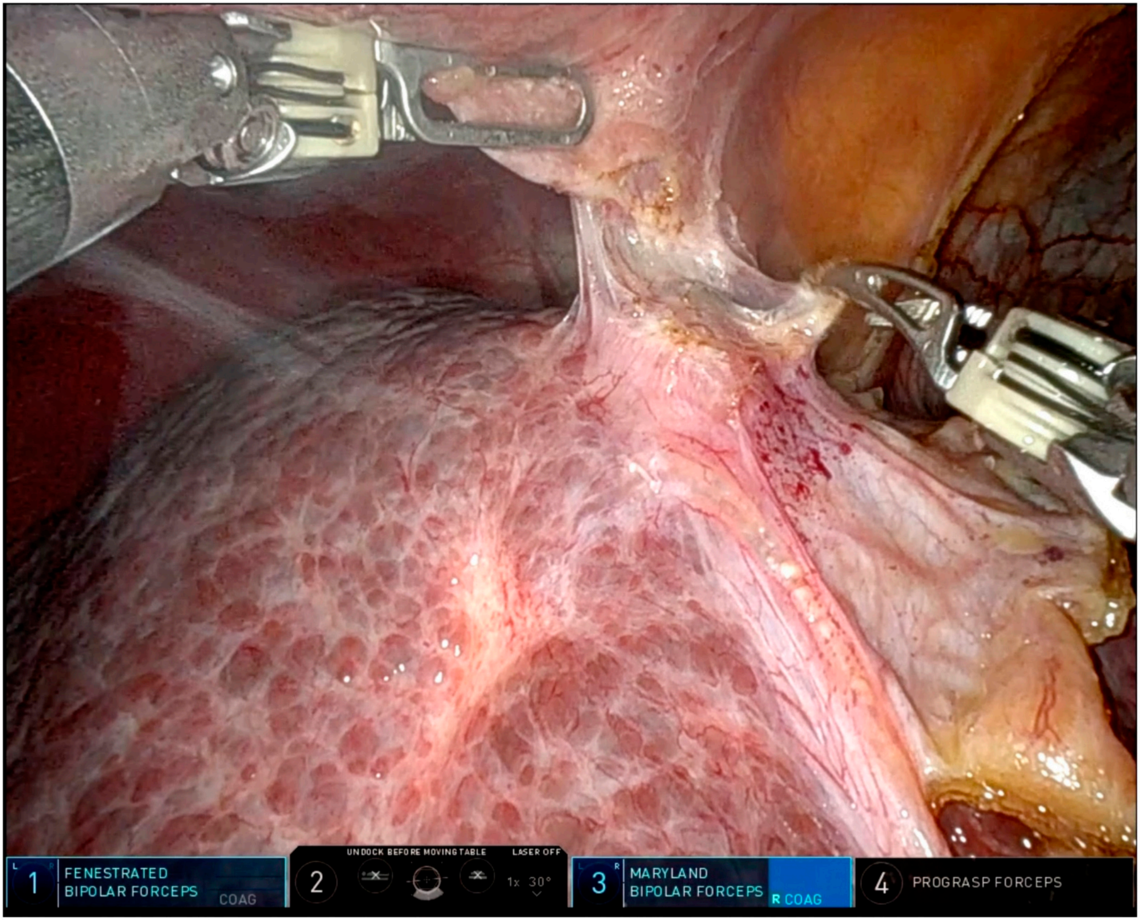

Supplementary Figure 1. Division of the falciform ligament of the recipient.

Pioneering Fully Robotic Donor Hepatectomy and Robotic Recipient Liver Graft Implantation – A New Horizon in Liver Transplantation

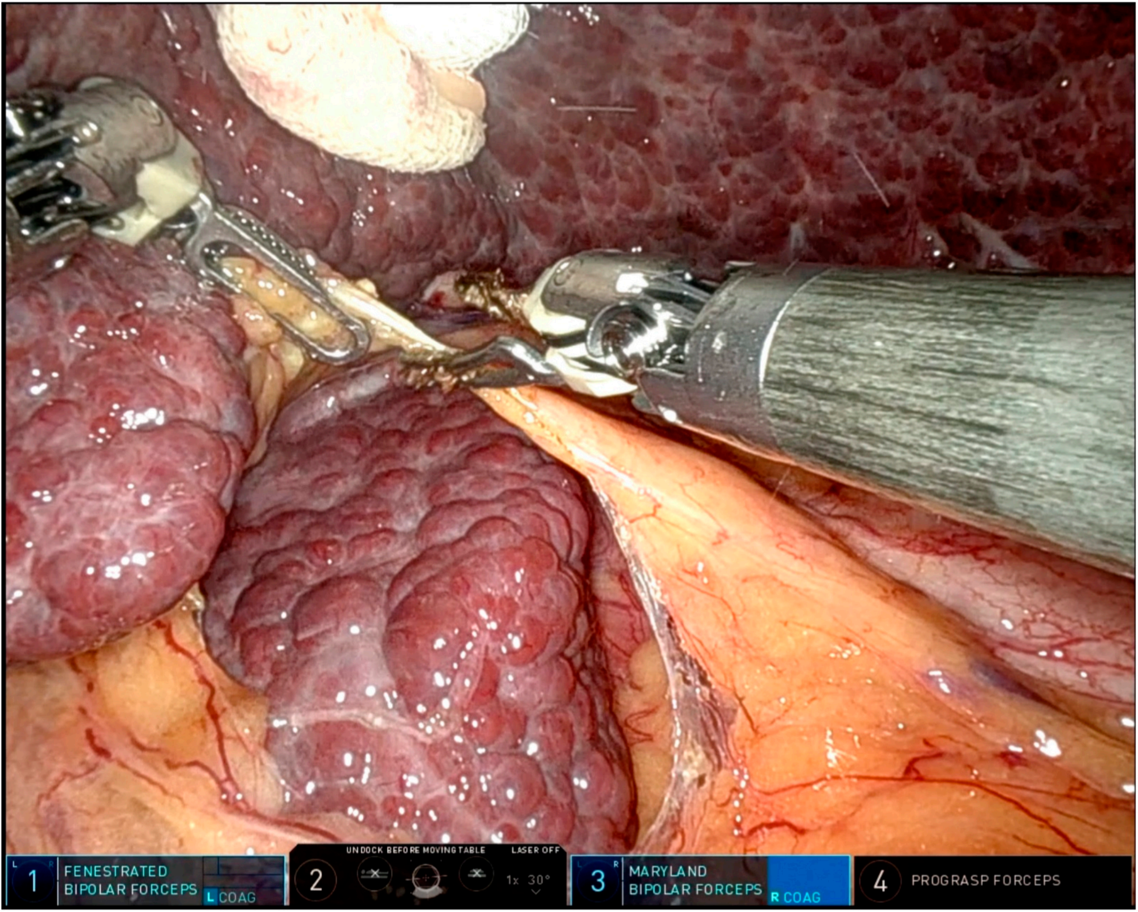

Supplementary Figure 2. Division of the gastrohepatic ligament of the recipient.

Pioneering Fully Robotic Donor Hepatectomy and Robotic Recipient Liver Graft Implantation – A New Horizon in Liver Transplantation

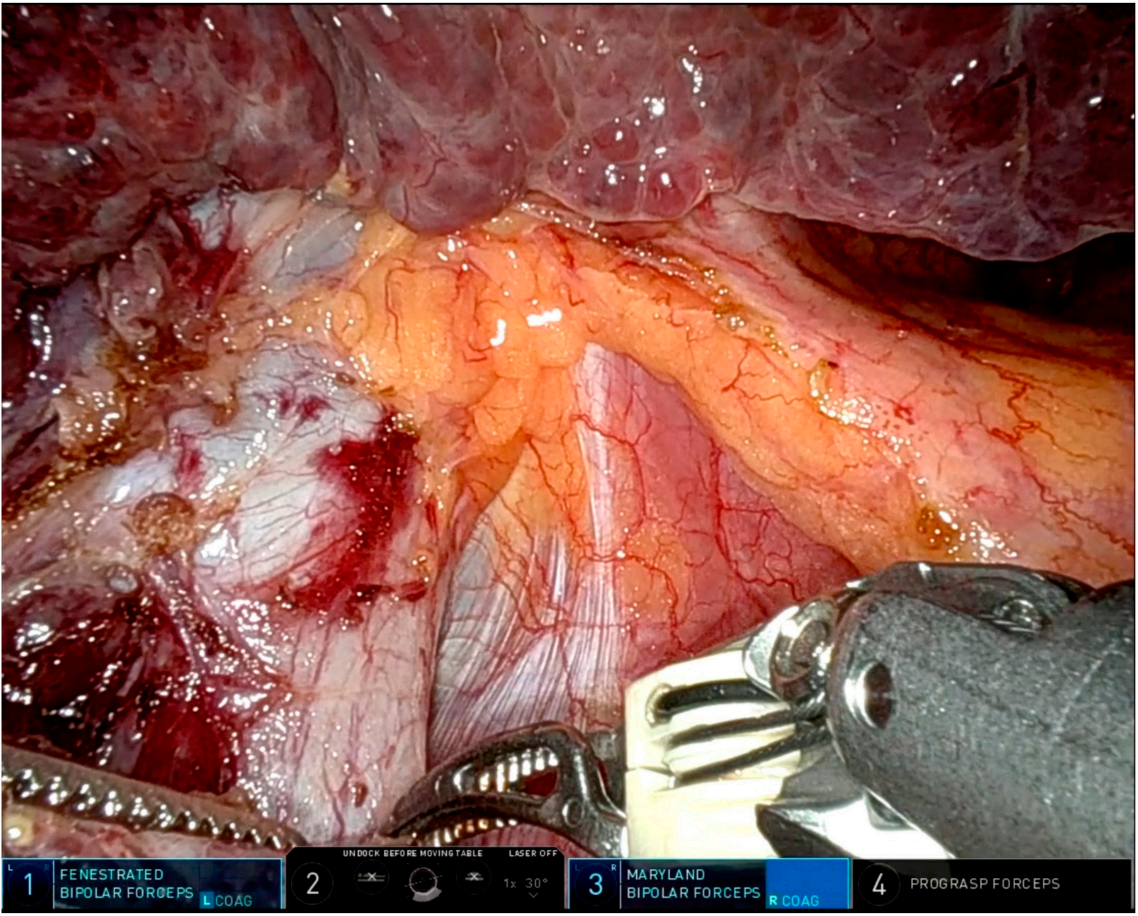

Supplementary Figure 3. Exposure of the left side of the retrohepatic inferior vena cava of the recipient.

Pioneering Fully Robotic Donor Hepatectomy and Robotic Recipient Liver Graft Implantation – A New Horizon in Liver Transplantation

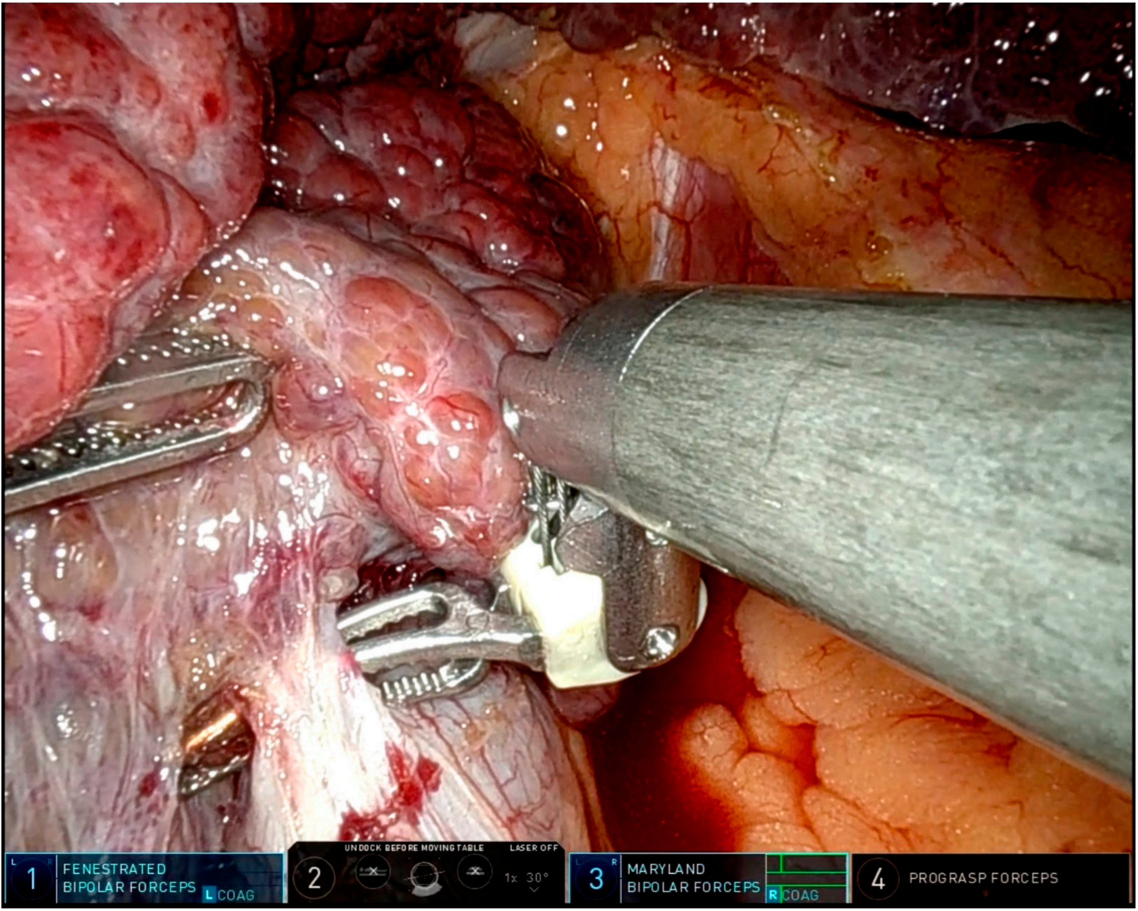

Supplementary Figure 4. Skeletisation of the segment 1 hepatic vein of the recipient.

Pioneering Fully Robotic Donor Hepatectomy and Robotic Recipient Liver Graft Implantation – A New Horizon in Liver Transplantation

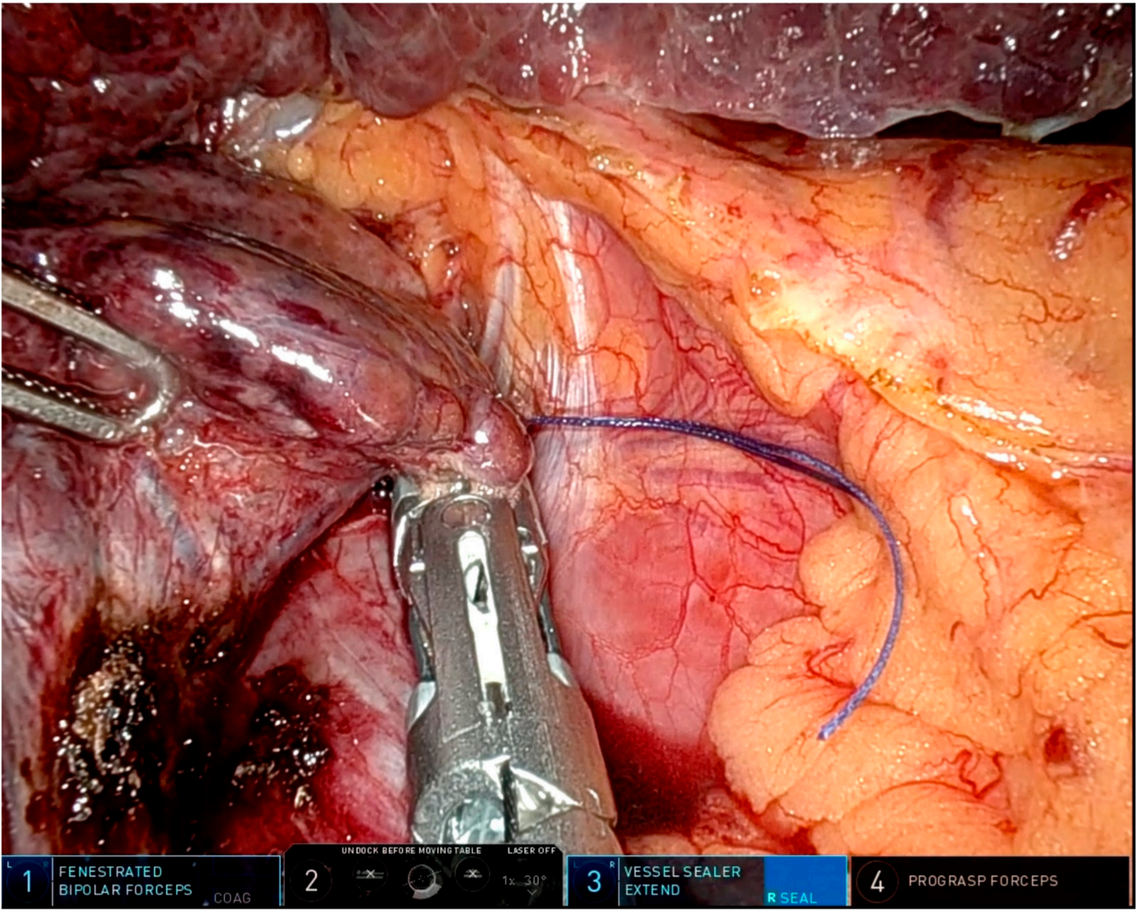

Supplementary Figure 5. Division of the segment 1 hepatic vein using the Vessel Sealer of the recipient.

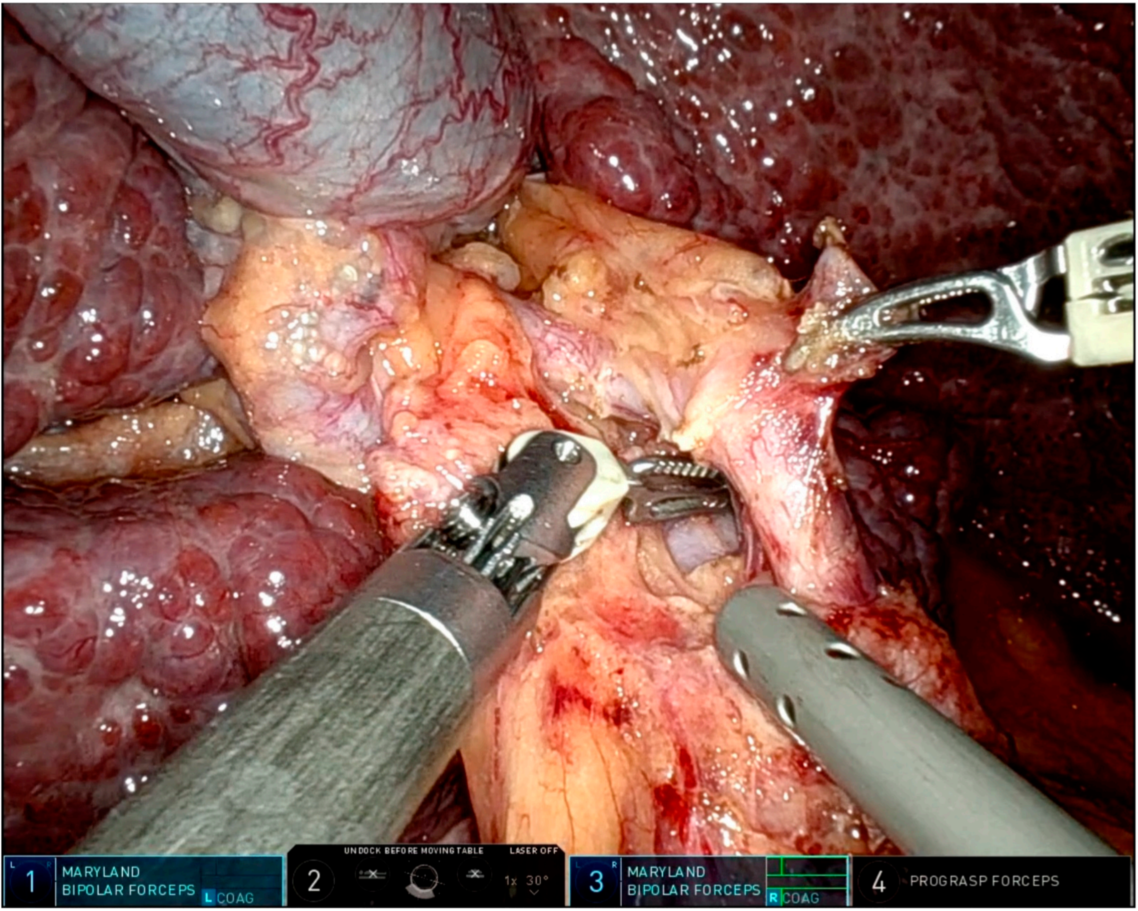

Supplementary Figure 6. Dissection of the porta hepatis and skeletonization of the hepatic artery of the recipient.

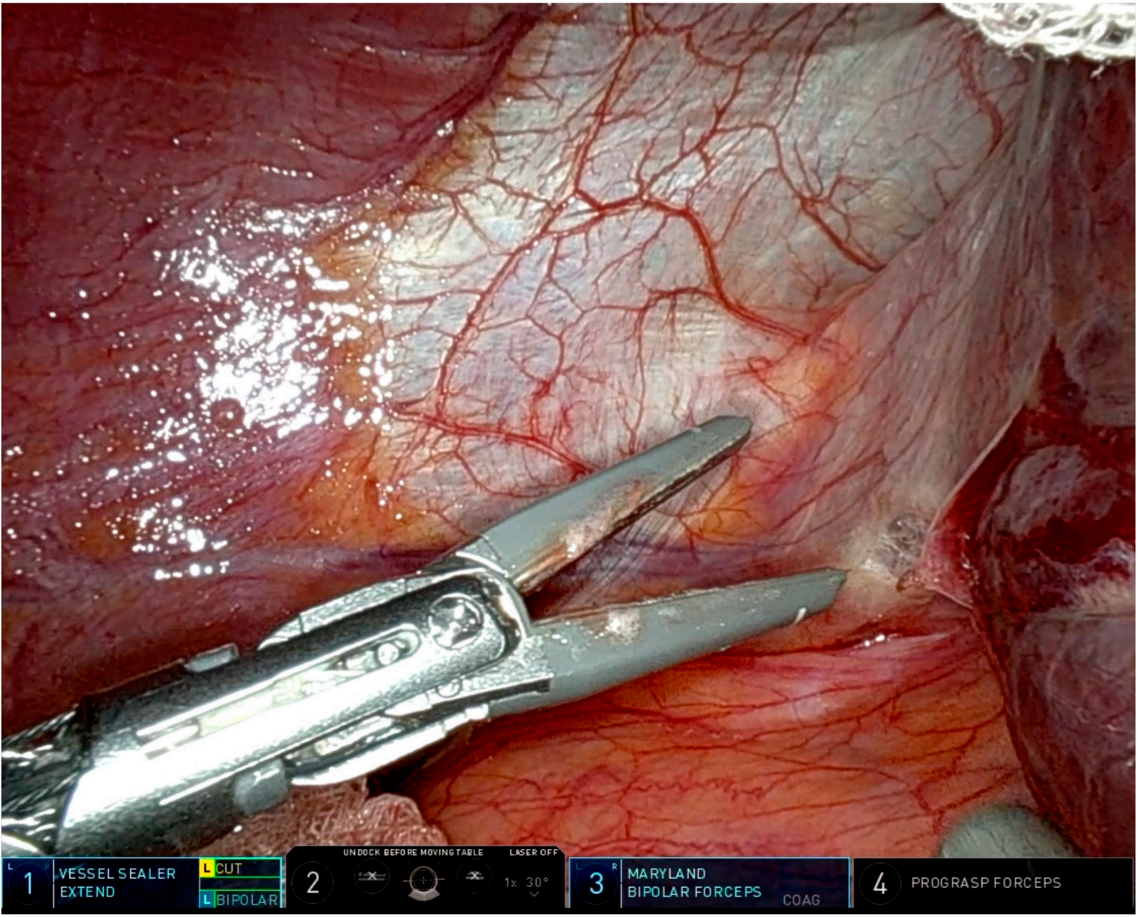

Supplementary Figure 7. Division of the right triangular ligament, mobilization of the right liver of the recipient.

Pioneering Fully Robotic Donor Hepatectomy and Robotic Recipient Liver Graft Implantation – A New Horizon in Liver Transplantation

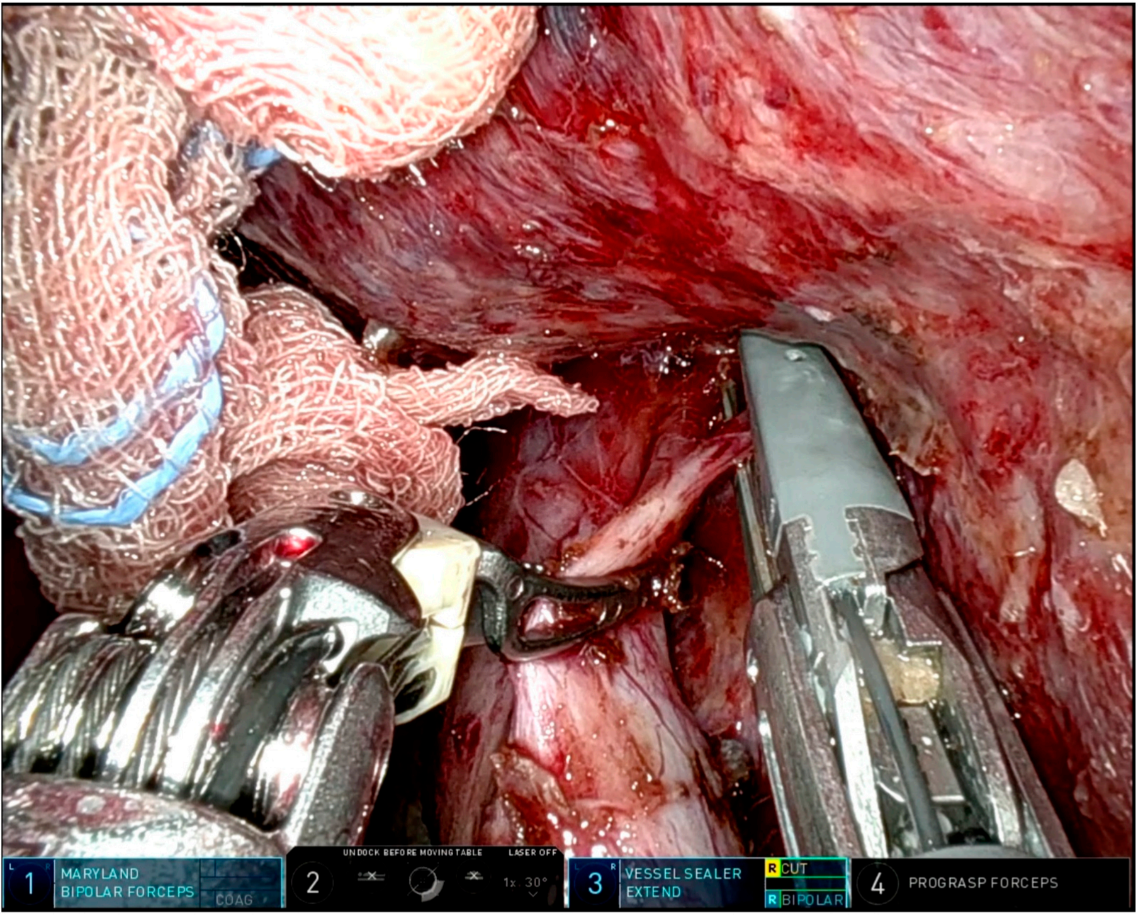

Supplementary Figure 8. Division of an inferior hepatic vein using the Vessel Sealer from the right side of the recipient.
