# Supplementary material for: Pioneering fully robotic donor hepatectomy and robotic recipient liver graft implantation – a new horizon in liver transplantation
Source: Int J Surg. 2024 Jan 4;110(3):1333–6. doi: 10.1097/JS9.0000000000001031 (PMC10942232; doi:10.1097/JS9.0000000000001031)
Supplement: SUPPLEMENTARY MATERIAL [file js9-110-1333-s002.pdf]

Pioneering Fully Robotic Donor Hepatectomy and Robotic Recipient Liver Graft Implantation – A New Horizon in Liver Transplantation

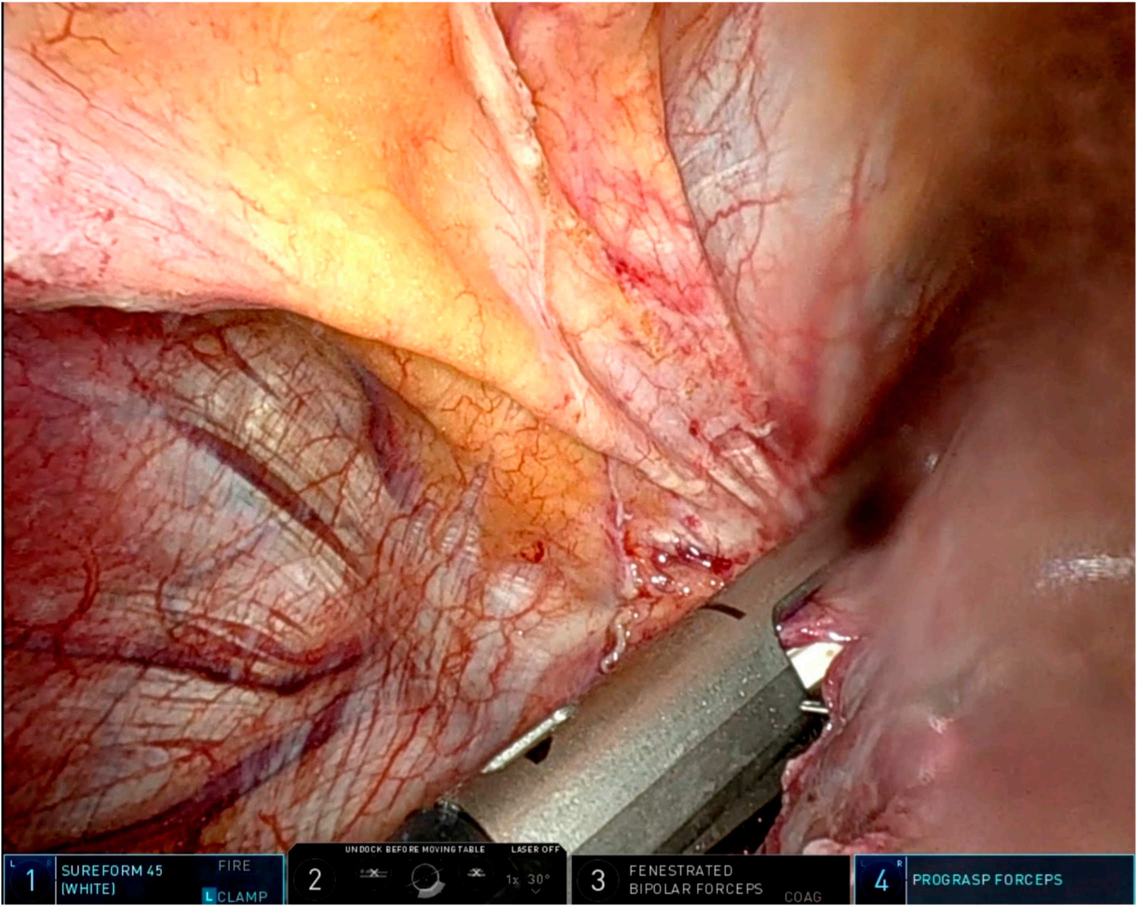

Supplementary Figure 9. Stapling the right hepatic vein of the recipient.

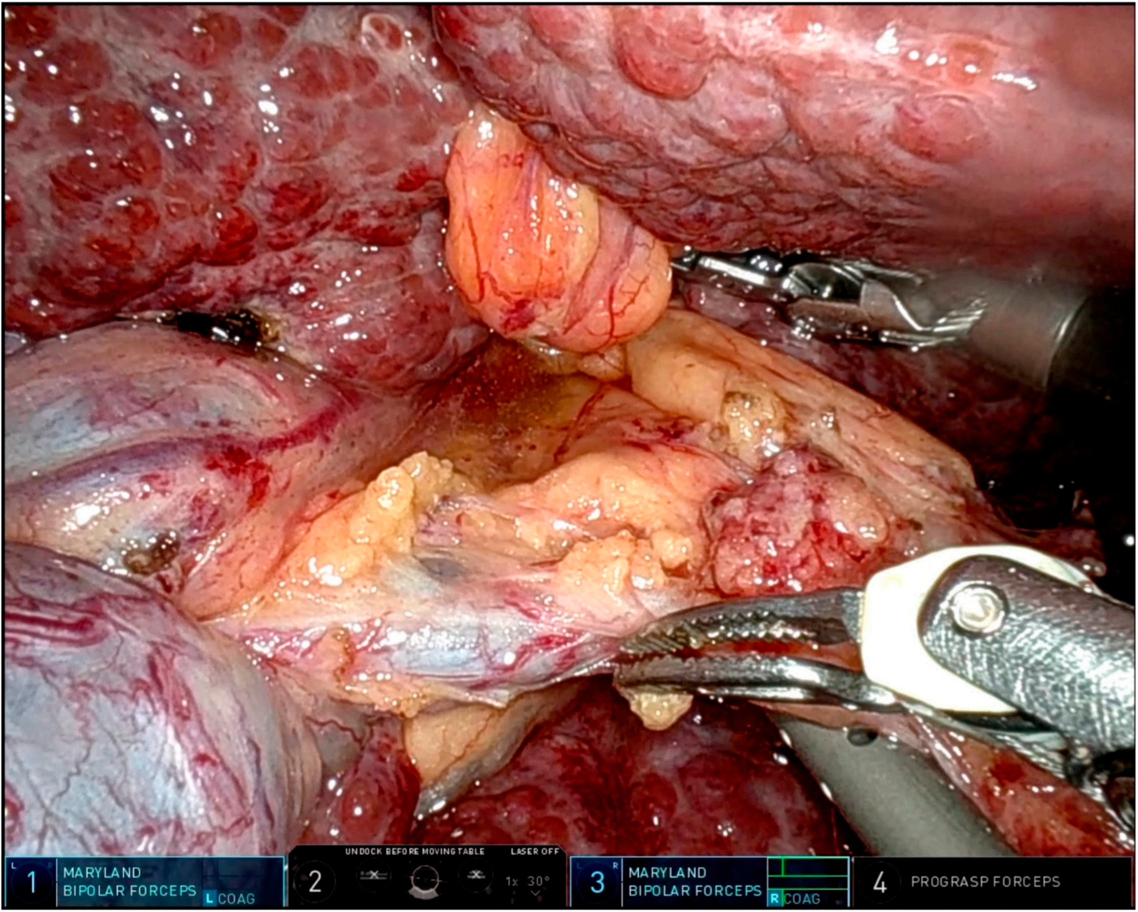

Supplementary Figure 10. Exposure of the recipient common bile duct.

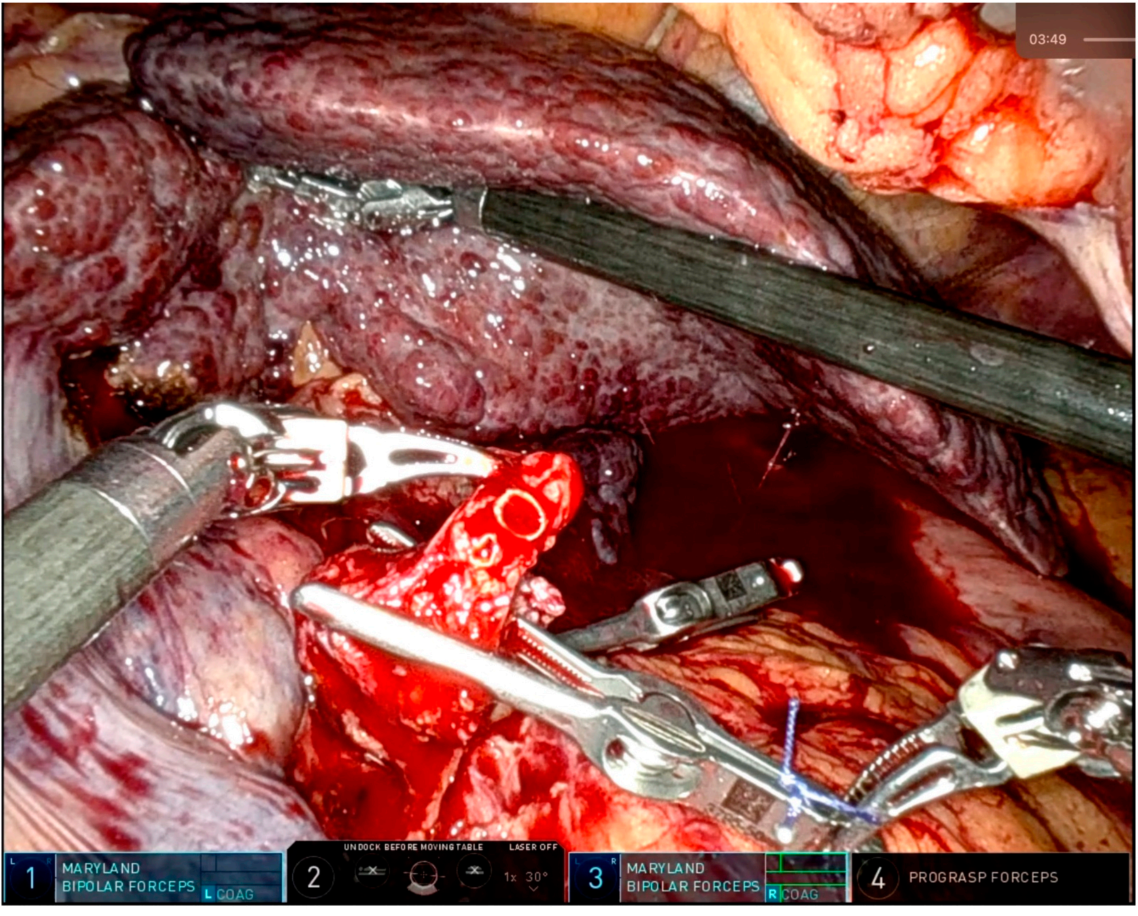

Supplementary Figure 11. Division of the recipient common hepatic duct and placement of a bulldog clamp.

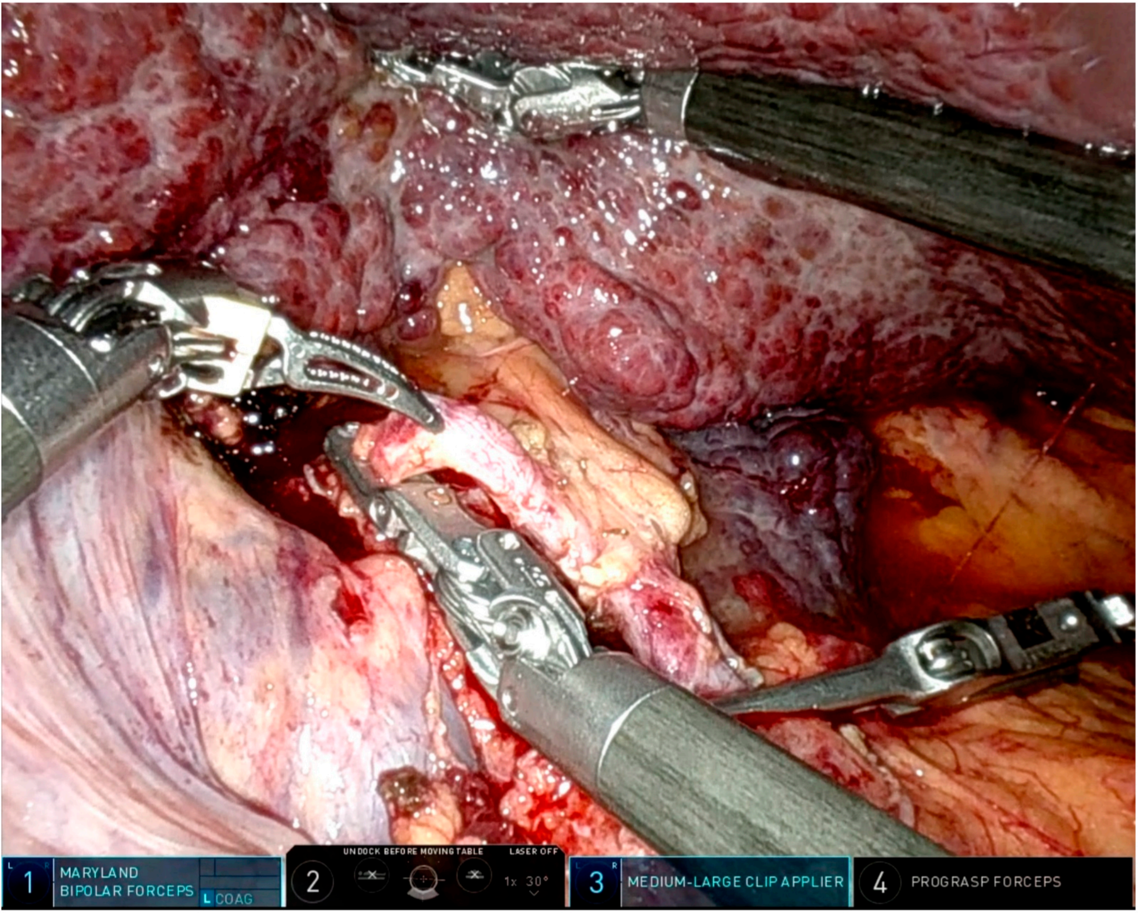

Supplementary Figure 12. Clamping distally with a bulldog clamp proximally with a hem-o-lok clip the hepatic artery of the recipient.

Pioneering Fully Robotic Donor Hepatectomy and Robotic Recipient Liver Graft Implantation – A New Horizon in Liver Transplantation

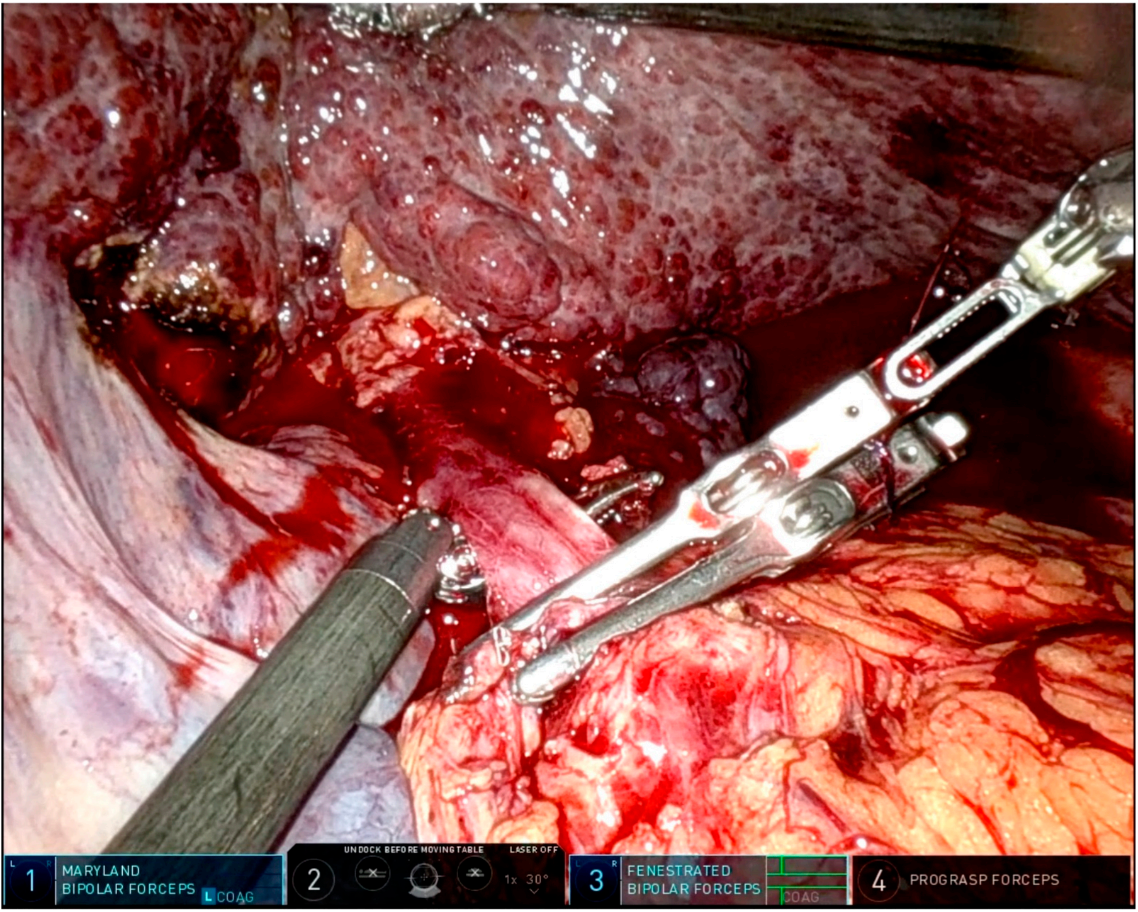

Supplementary Figure 13. Clamping distally with two bulldog clamps the portal vein of the recipient prior to division.

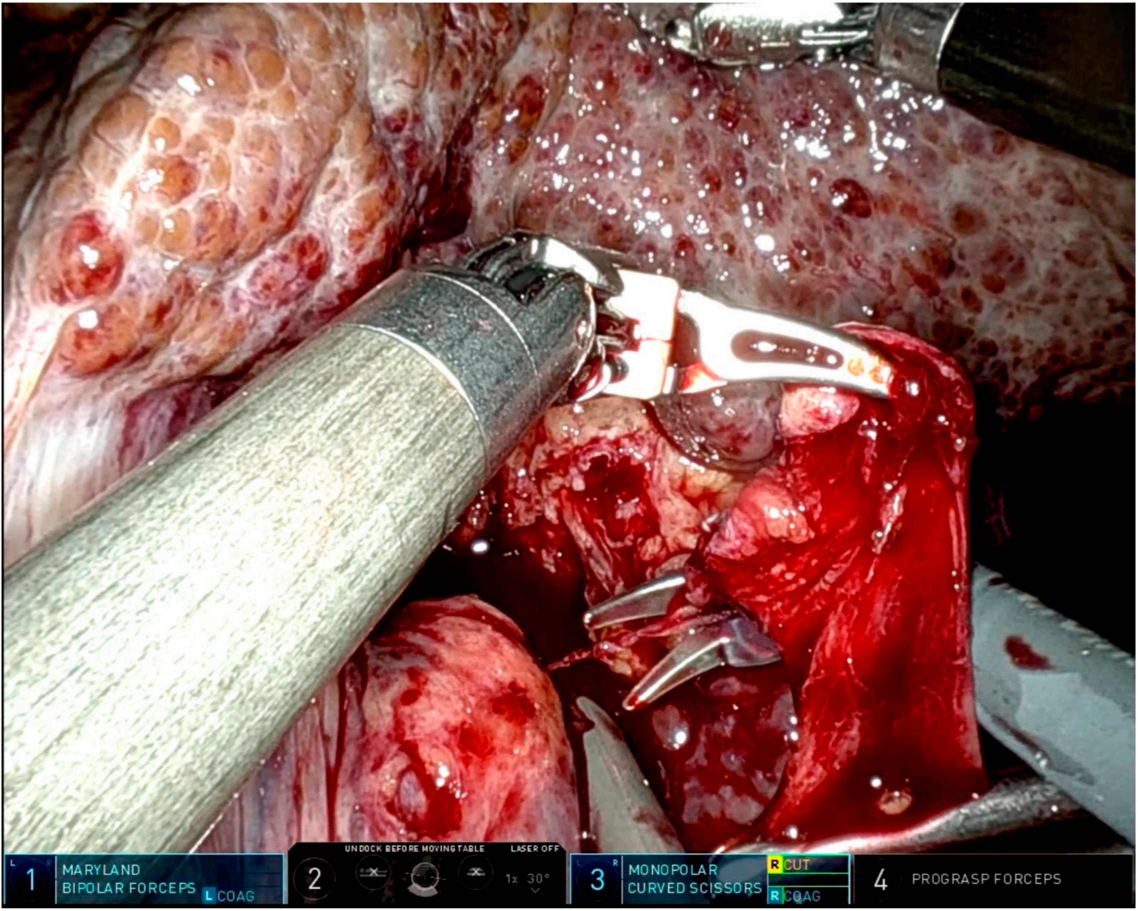

Supplementary Figure 14. Division of the recipient portal vein with scissors proximally at the hilum.

Pioneering Fully Robotic Donor Hepatectomy and Robotic Recipient Liver Graft Implantation – A New Horizon in Liver Transplantation

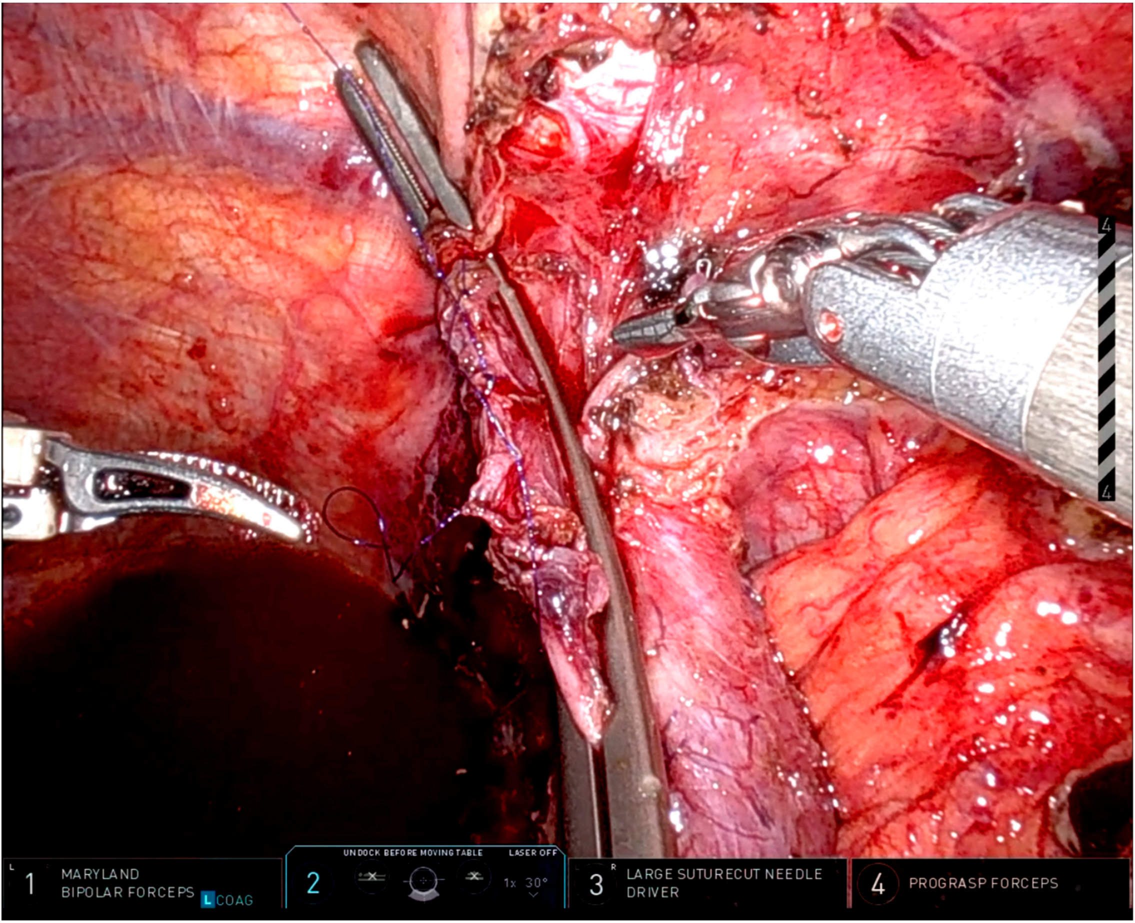

Supplementary Figure 15. Partial clamping of the recipient inferior vena cava beneath the right hepatic vein staple line with an intracorporeal Glover Clamp.
