# Supplementary material for: Pioneering fully robotic donor hepatectomy and robotic recipient liver graft implantation – a new horizon in liver transplantation
Source: Int J Surg. 2024 Jan 4;110(3):1333–6. doi: 10.1097/JS9.0000000000001031 (PMC10942232; doi:10.1097/JS9.0000000000001031)
Supplement: SUPPLEMENTARY MATERIAL [file js9-110-1333-s003.pdf]

Pioneering Fully Robotic Donor Hepatectomy and Robotic Recipient Liver Graft Implantation – A New Horizon in Liver Transplantation

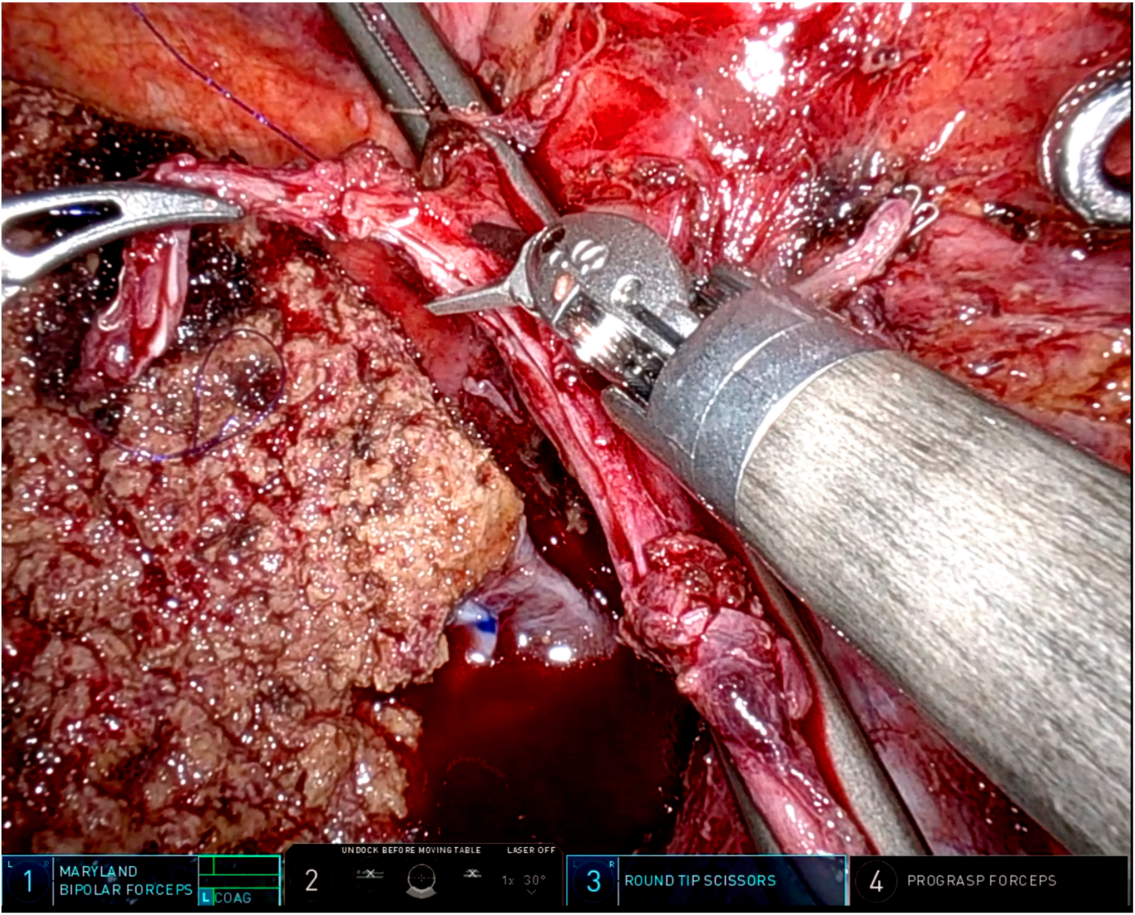

Supplementary Figure 16. Excision of the recipient right hepatic vein staple line.

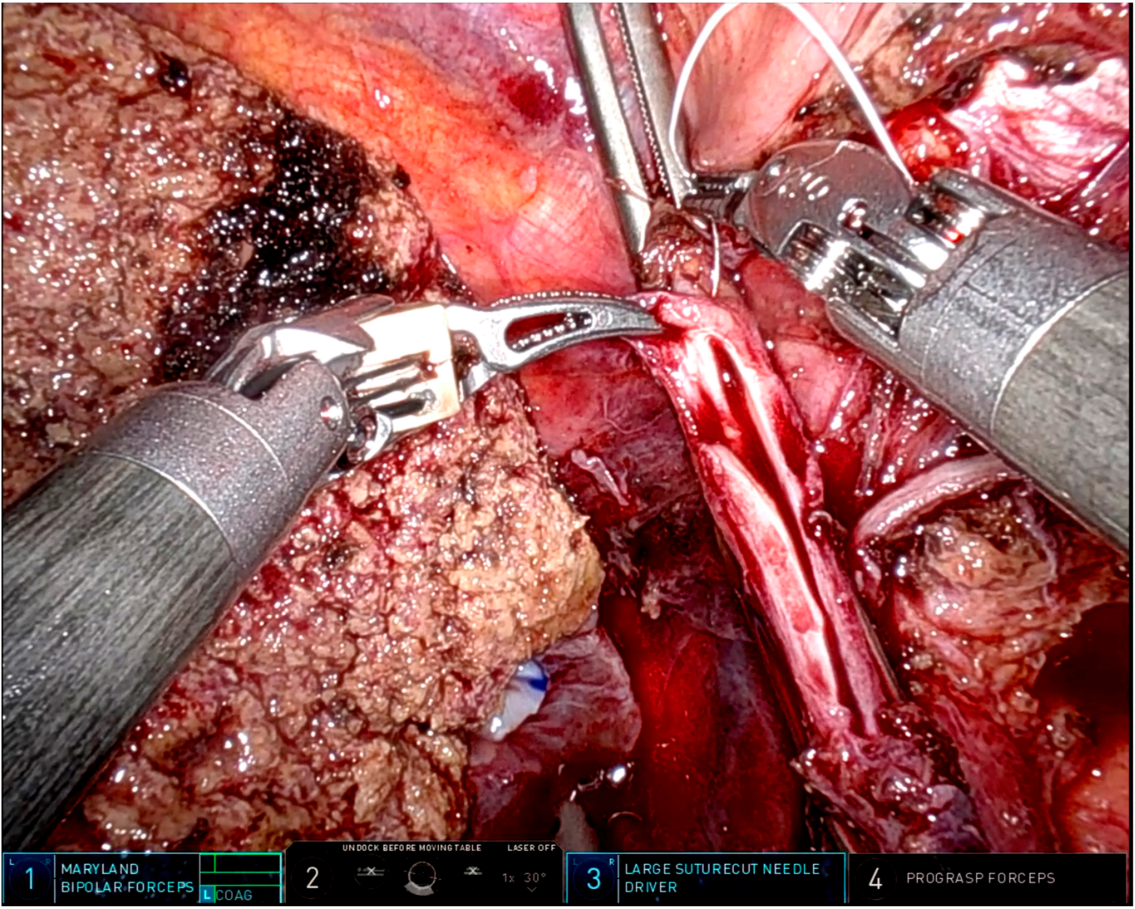

Supplementary Figure 17. Insertion of the right donor graft and start of the right hepatic vein anastomosis in a continuous fashion using a Gortex 6/0 suture.

Pioneering Fully Robotic Donor Hepatectomy and Robotic Recipient Liver Graft Implantation – A New Horizon in Liver Transplantation

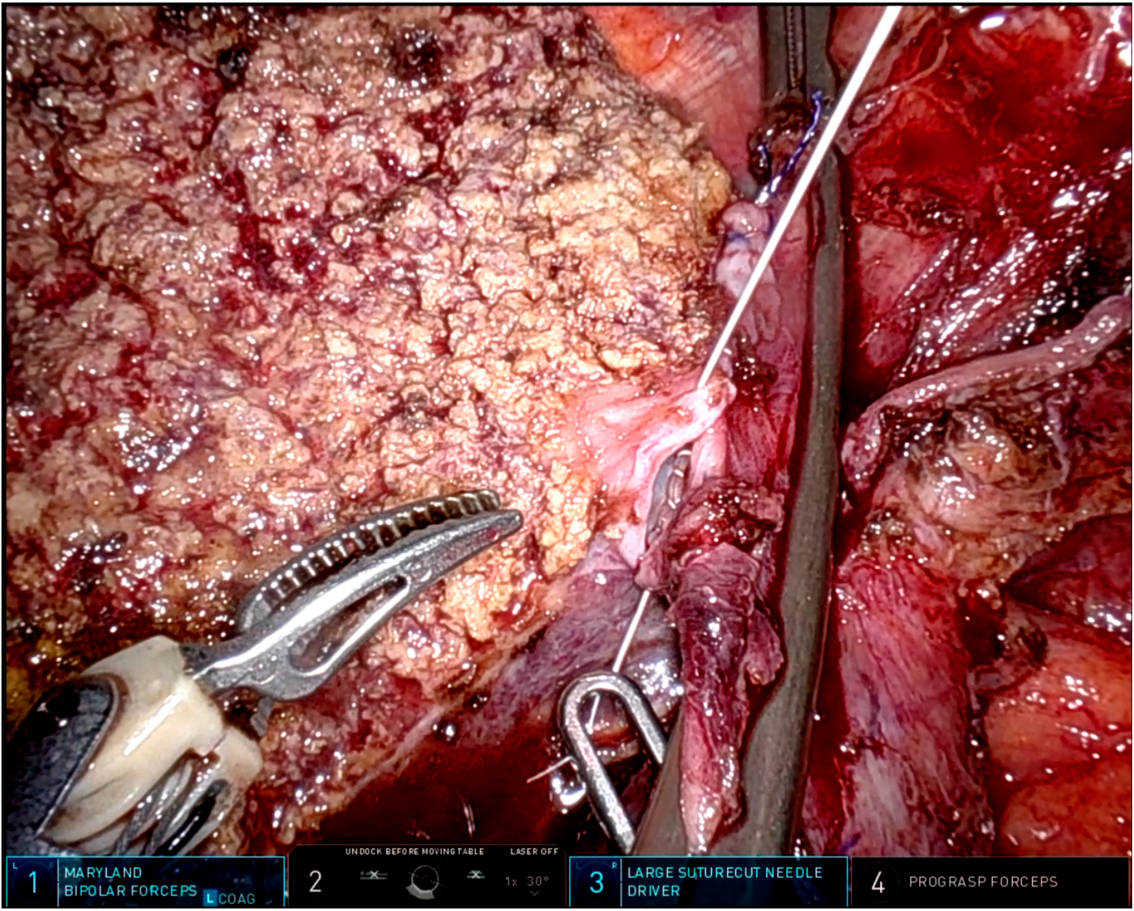

Supplementary Figure 18. Completion of the right hepatic vein anastomosis back and front walls.

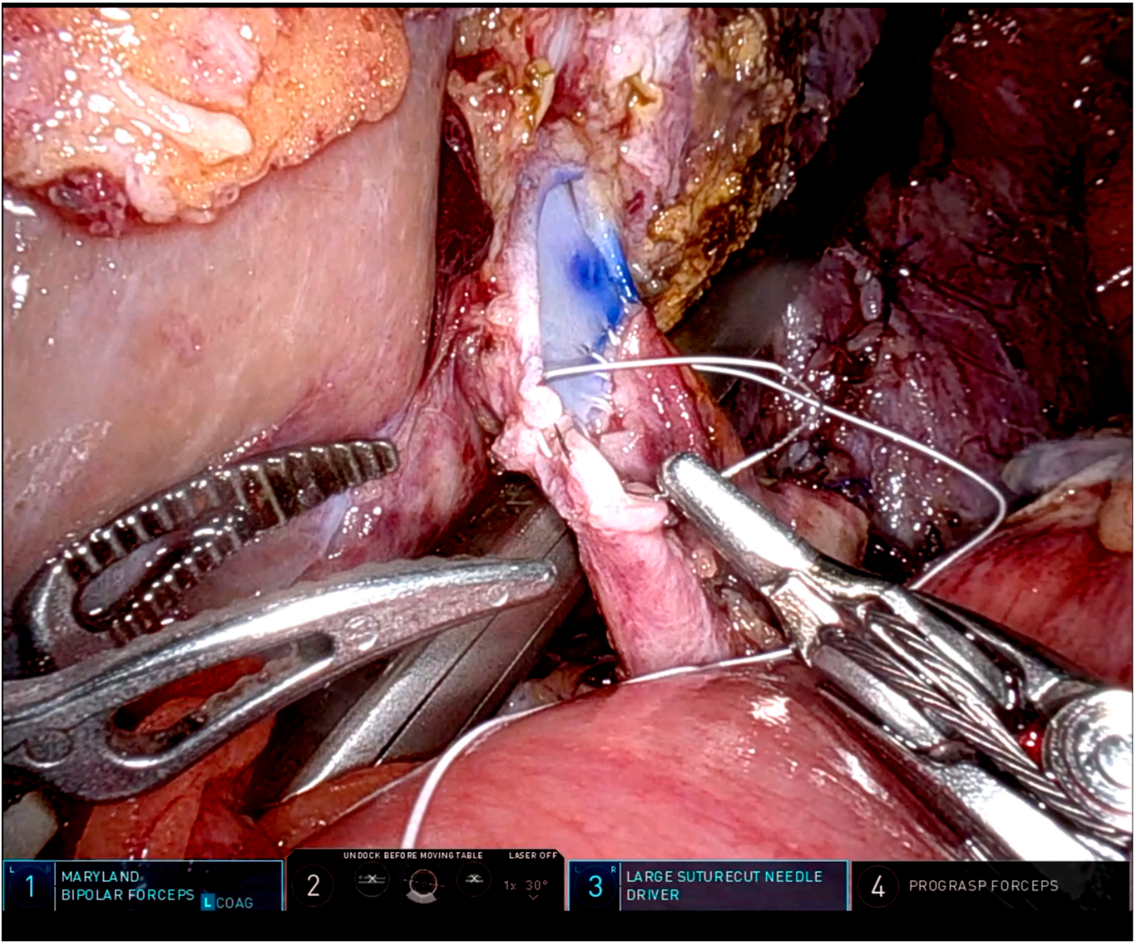

Supplementary Figure 19. Start of the right portal vein anastomosis back wall using a continuous Gortex 6/0 suture.

Pioneering Fully Robotic Donor Hepatectomy and Robotic Recipient Liver Graft Implantation – A New Horizon in Liver Transplantation

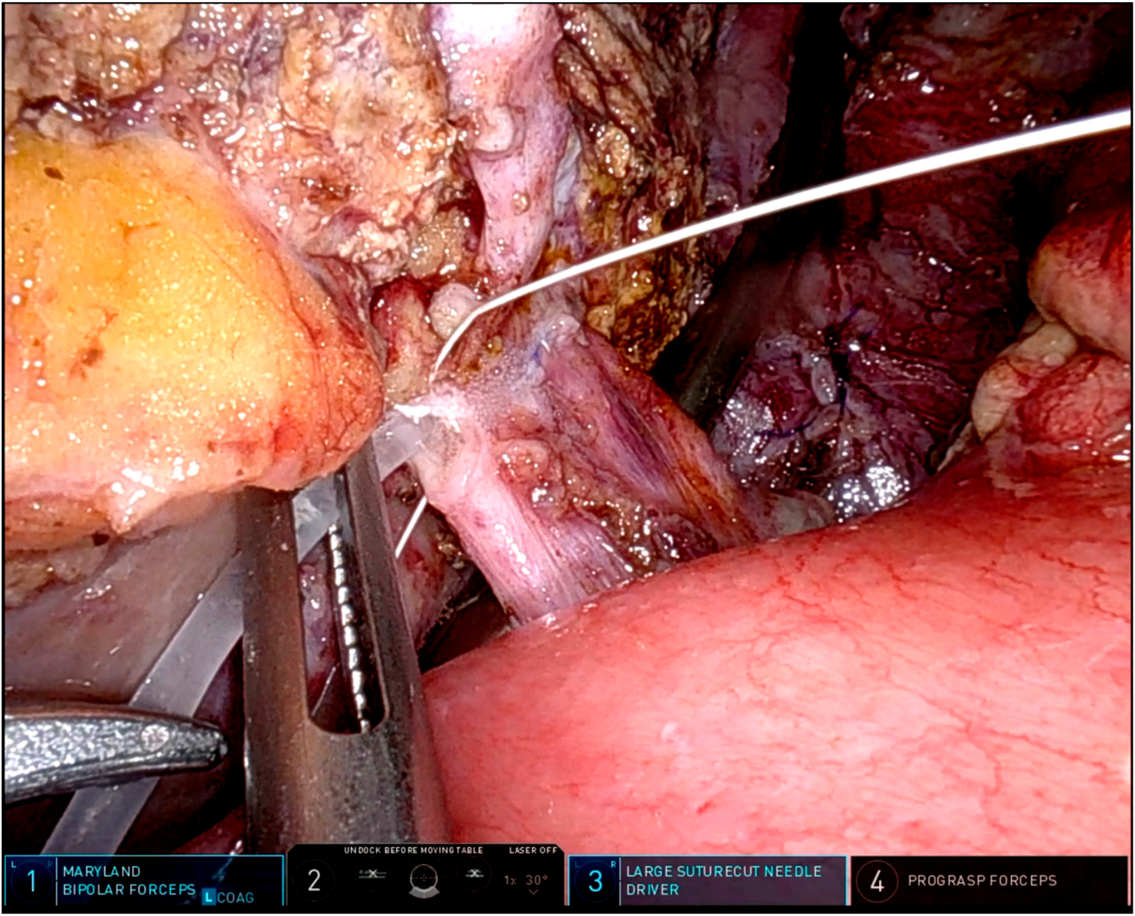

Supplementary Figure 20. Completion of the right portal vein anastomosis and flushing.
