# Supplementary material for: Pioneering fully robotic donor hepatectomy and robotic recipient liver graft implantation – a new horizon in liver transplantation
Source: Int J Surg. 2024 Jan 4;110(3):1333–6. doi: 10.1097/JS9.0000000000001031 (PMC10942232; doi:10.1097/JS9.0000000000001031)
Supplement: SUPPLEMENTARY MATERIAL [file js9-110-1333-s004.pdf]

Pioneering Fully Robotic Donor Hepatectomy and Robotic Recipient Liver Graft Implantation – A New Horizon in Liver Transplantation

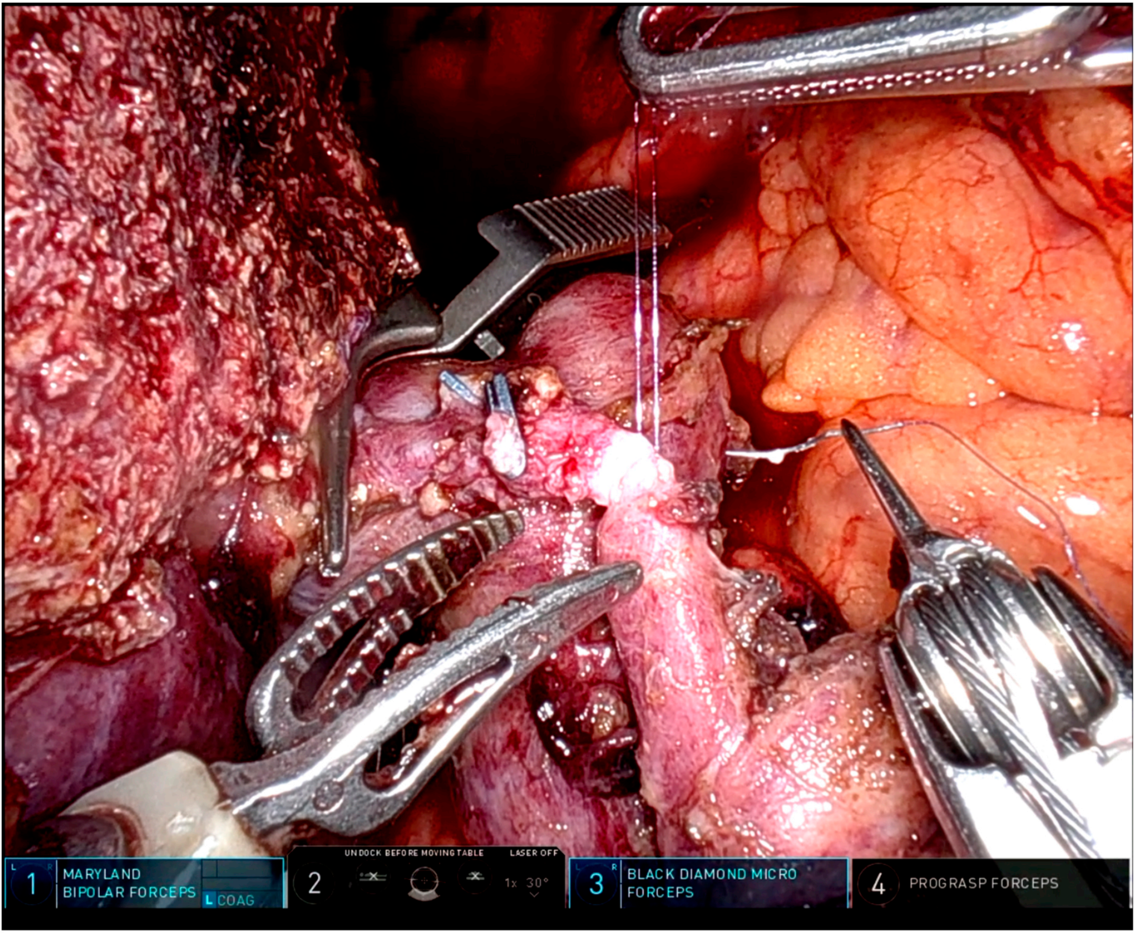

Supplementary Figure 21. Start of the hepatic artery anastomosis with interrupted 7/0 Prolene sutures.

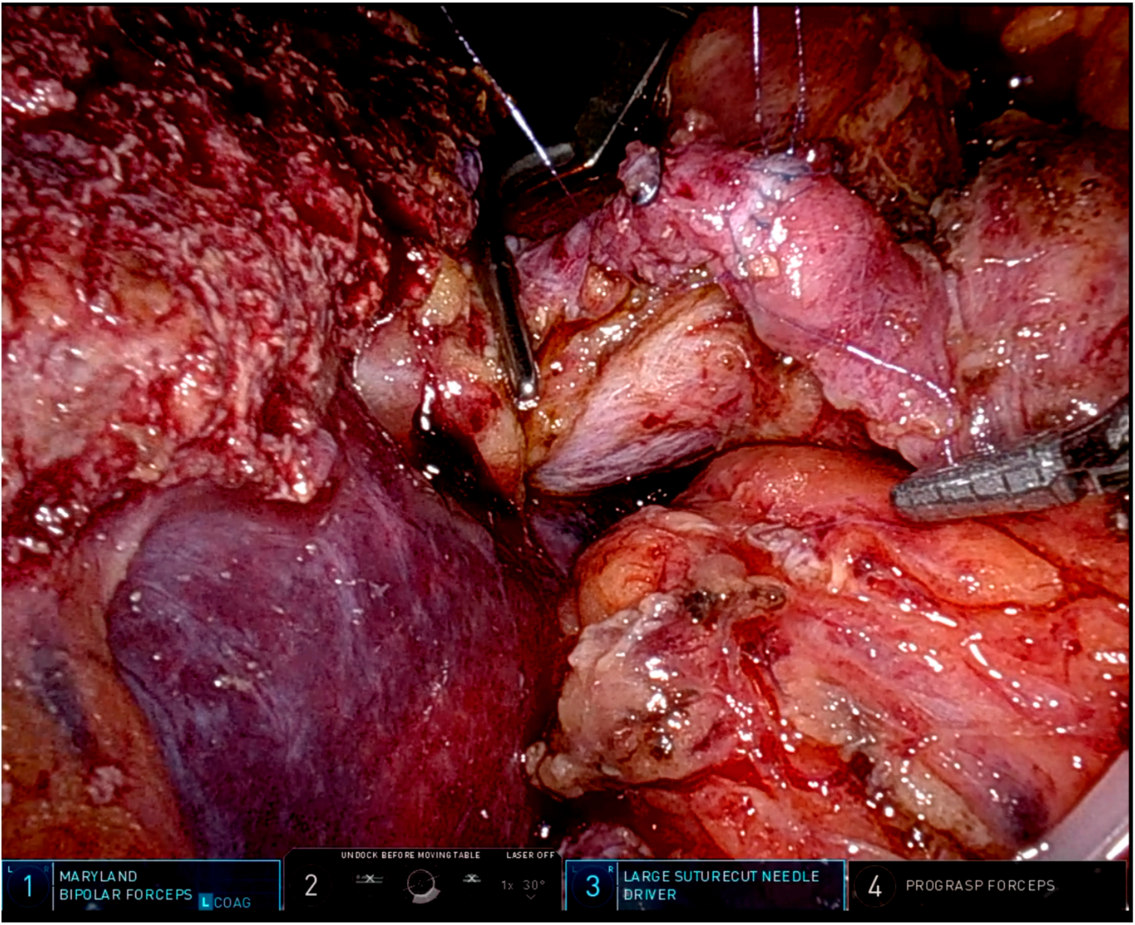

Supplementary Figure 22. Completion of the hepatic artery anastomosis with interrupted 7/0 Prolene sutures.

Pioneering Fully Robotic Donor Hepatectomy and Robotic Recipient Liver Graft Implantation – A New Horizon in Liver Transplantation

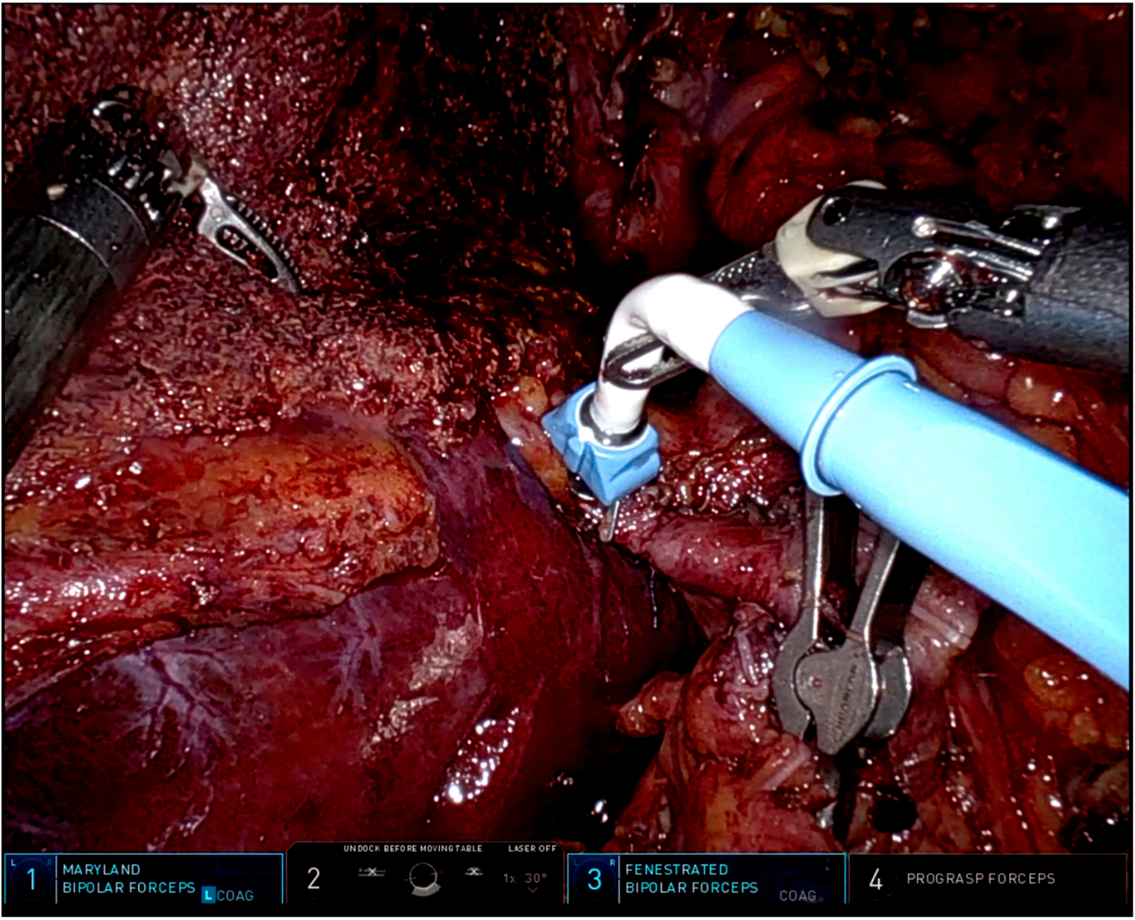

Supplementary Figure 23. Hepatic artery volume flow measurement.

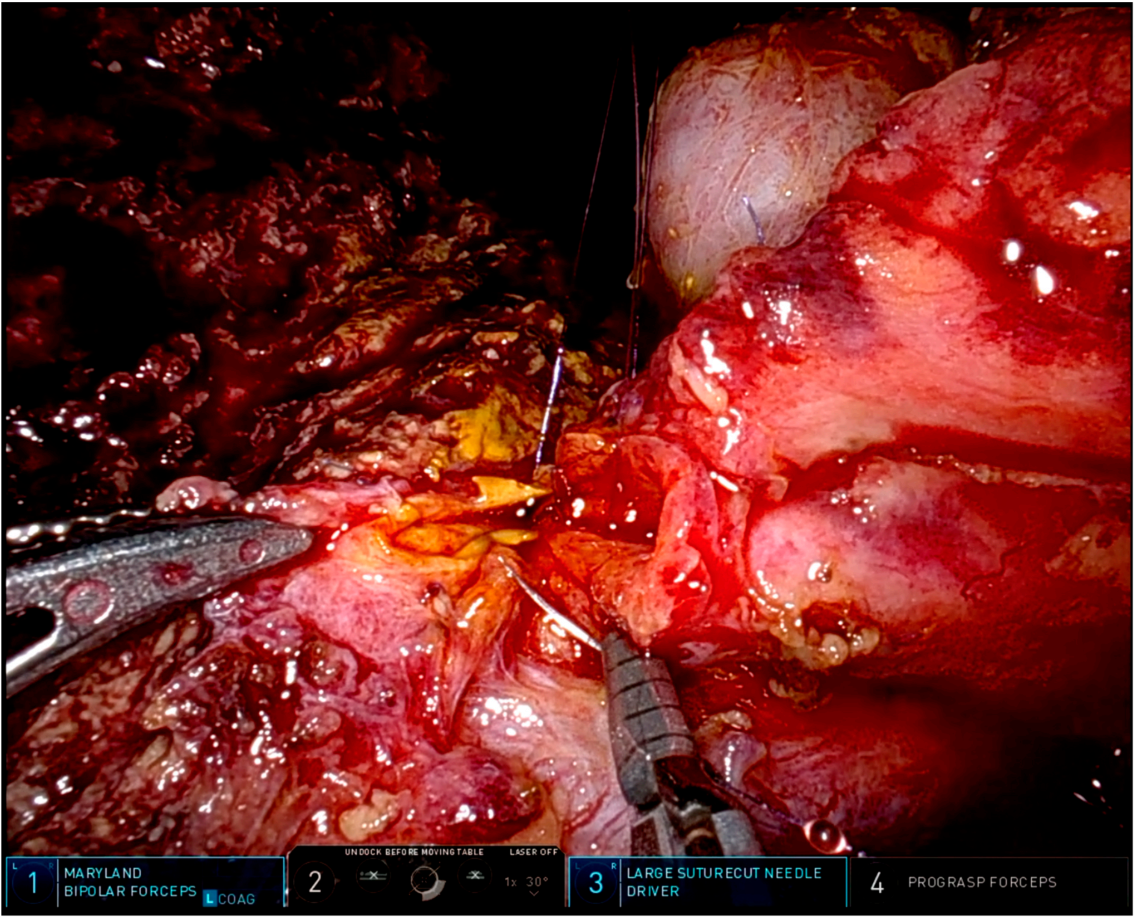

Supplementary Figure 24. Start of the duct-to-duct anastomosis using interrupted 7/0 PDS.

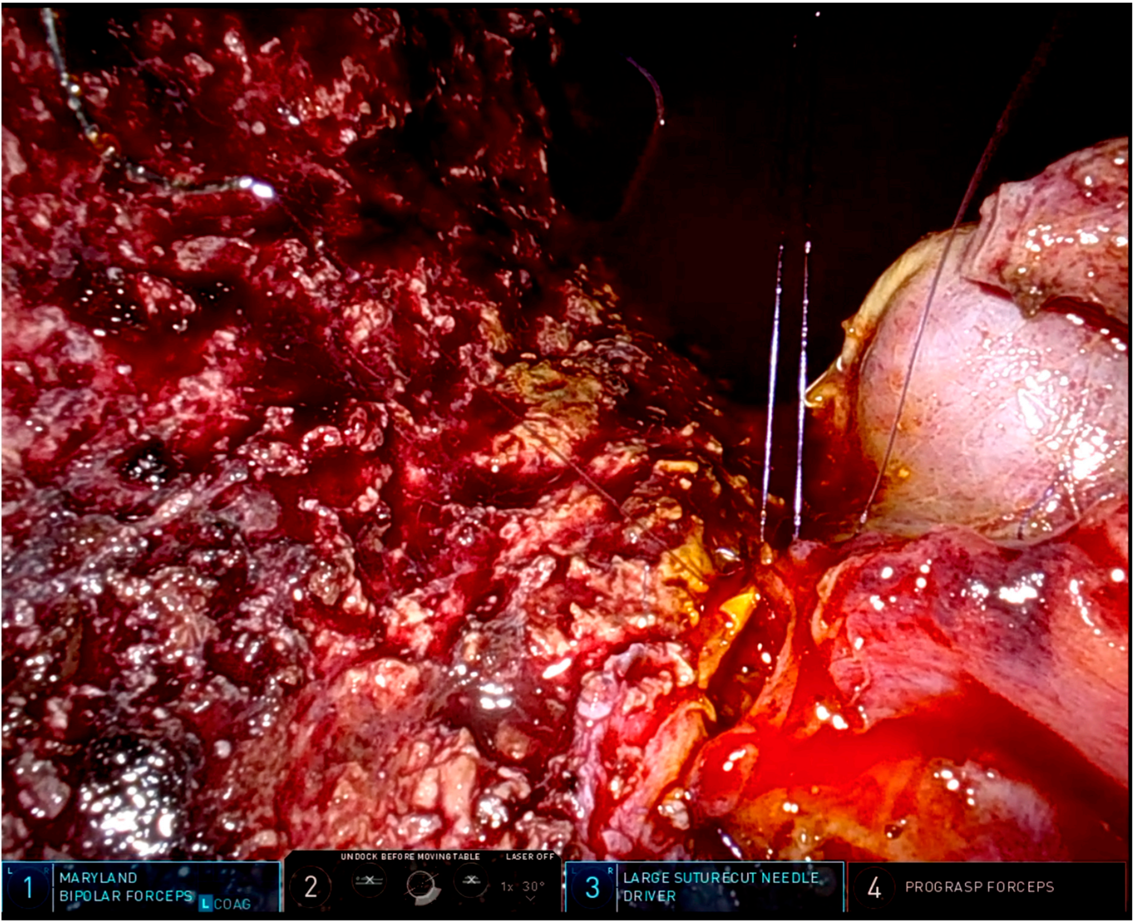

Supplementary Figure 25. Completion of the back wall and start of front wall of the duct-to-duct anastomosis.

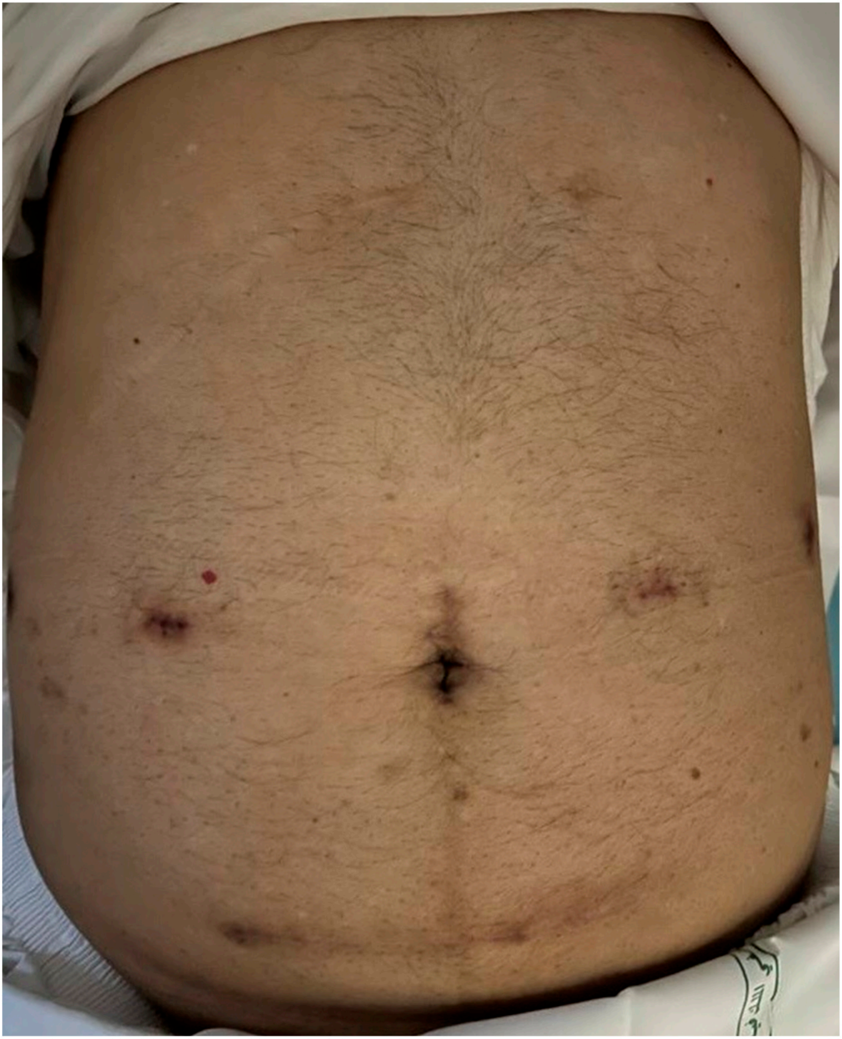

Supplementary Figure 26. Photograph of the recipient’s abdomen 30 days post robotic liver transplantation
